# Associations of *IL*-*10* genetic polymorphisms with the risk of urologic cancer: a meta-analysis based on 18,415 subjects

**DOI:** 10.1186/s40064-016-3705-0

**Published:** 2016-11-29

**Authors:** Xiaohan Shi, Xiaochuan Xie, Xiaoshuang Xun, Yingxian Jia, Shangwei Li

**Affiliations:** 1 Division of Reproductive Medical Center, West China Second University Hospital, Sichuan University, Chengdu, Sichuan China; 2 Key Laboratory of Birth Defects and Related Diseases of Women and Children, West China Second University Hospital of Sichuan University, Chengdu, Sichuan China; 3 Department of Cardiology, West China Hospital, Sichuan University, Chengdu, Sichuan China; 4 West China School of Public Health, Sichuan University, Chengdu, Sichuan China

**Keywords:** Interleukin-10 (IL-10), Urologic cancer, Genetic polymorphisms, Meta-analysis

## Abstract

**Background:**

Interleukin-10 (IL-10) is a powerful modulator of anti-tumor immune responses. The *IL*-*10* promoter region polymorphisms are known to regulate IL-10 production, and thus are thought to be implicated in tumorigenesis. Recently, the roles of these polymorphisms in urologic cancer have been extensively studied, with conflicting results. Therefore, we conducted the present meta-analysis to better elucidate the correlations between *IL*-*10* polymorphisms and urologic cancer risk.

**Methods:**

Eligible articles were searched in PubMed, Medline, Embase, Scopus and CNKI up to May 2016. Odds ratios (ORs) and 95% confidence intervals (CIs) were used to detect any potential associations between *IL*-*10* polymorphisms and the risk of urologic cancer.

**Results:**

A total of 22 case–control studies including 8572 patients and 9843 controls were analyzed. The overall meta-analysis results showed that *IL*-*10* −592C>A polymorphism was significantly associated with urologic cancer in CA versus AA (*P* = 0.04, OR 0.87, 95% CI 0.76–0.99) and AA versus CC+CA (*P* = 0.03, OR 1.15, 95% CI 1.02–1.31). Subgroup analyses by cancer types suggested there were significant associations between all the three investigated *IL*-*10* polymorphisms and bladder cancer. However, subgroup analyses by ethnicity only detected a weak association between *IL*-*10* −819C>T and Asian population.

**Conclusions:**

Our findings suggests that *IL*-*10* −592C>A polymorphism may implicate with urologic cancer risk. Besides, promoter region polymorphisms of *IL*-*10* may serve as potential biological markers, especially for bladder cancer. Furthermore, *IL*-*10* −819C>T polymorphism may contribute to urologic cancer susceptibility in Asians while all the three studied variants of *IL*-*10* did not relate to Caucasian urologic cancer predisposition.

## Background

Commonly seen urologic cancers such as prostate cancer, renal cancer, and bladder cancer are leading causes of cancer-related morbidity and mortality globally (Siegel et al. 2014). Despite rapid advances in early diagnosis and surgical treatment over the past few decades, the numbers of new urologic cancer cases and associated deaths are continue to increase, making it becomes one of the major threats to public health worldwide (Ferlay et al. 2015).

To date, the exact cause of urologic cancer remains unclear. Certain environmental factors like smoking habit, heavy alcohol intake, high caloric diet and chemical dyes have been identified as potential etiological factors for urologic cancer. However, the fact that only a small portion of individuals exposed to these carcinogenic agents ultimately develop urologic cancer suggests that genetic susceptibility factors may play a crucial part in its pathogenesis (Jiang et al. 2014).

Interleukin-10 (IL-10), encoded by the *IL*-*10* gene located on chromosome 1q31–32, is a potent regulator of anti-tumor immune responses (Eskdale et al. 1997; Mocellin et al. 2005). As a result, certain polymorphisms located in the promoter region of *IL*-*10* gene (−592C>A, −819C>T and −1082A>G), which regulate the expression level of IL-10 protein (Turner et al. 1997; Kingo et al. 2005), were thought to be implicated in the pathogenesis of various kinds of cancers. Recently, many genetic association studies have been carried out to investigate the potential correlations between *IL*-*10* promoter region polymorphisms and urologic cancer risk. However, results of these studies were controversial and the statistical power of individual studies was insufficient. Therefore, we conducted the present meta-analysis to better assess the potential associations of *IL*-*10* genetic polymorphisms with the risk of urologic cancer.

## Methods

### Literature searching strategy

To retrieve all relevant articles, a systematic literature search of PubMed, Medline, Embase, Scopus and China National Knowledge Infrastructure (CNKI) was performed using the following keywords: “Interleukin-10”, “IL-10”, “Interleukin 10”, “IL 10”, “polymorphism”, “variant”, “genotype”, “allele”, “prostate”, “renal”, “bladder”, “urinary”, “urologic”, “cancer”, “tumor”, “carcinoma”, “neoplasm” and “malignancy”. The initial search was conducted in September 2015 and the latest update was performed in May 2016. In addition, the reference lists of all retrieved articles were reviewed manually for further identification of potentially relevant articles.

### Inclusion criteria

The inclusion criteria for the present study were set prior to the literature search. Eligible studies met all the following conditions: (1) case–control study of unrelated urologic cancer patients and control subjects; (2) evaluation of the associations between *IL*-*10* polymorphisms (−592C>A, −819C>T and −1082A>G) and the risk of urologic cancer; (3) presentation of sufficient data to calculate the odds ratios (ORs) and corresponding 95% confidence intervals (CIs); (4) full text in English or Chinese available. If the report was duplicated or identical patients were enrolled in two studies, only the most recent and complete article was included. Abstracts, family-based association studies, case reports, case series, reviews, editorials, expert opinions and conference presentations were intentionally excluded.

### Data extraction and quality assessment

From each included studies, the following data were extracted: references, country of origin, ethnicity of study population, the number of cases and controls, types of urologic cancer, allelic and genotypic frequencies of *IL*-*10* polymorphisms in urologic cancer patients and control subjects, and whether the distributions of *IL*-*10* polymorphisms in the control group were in accordance with Hardy–Weinberg equilibrium (HWE). The Newcastle-ottawa quality assessment scale (NOS), a classical rating tool which evaluates the credibility of observational studies from three perspectives: selection, comparability and exposure, was used to assess the reliability of all case–control studies included (Zhang et al. 2014). This rating system has a score range of 0–9, and studies with a score of more than 7 were assumed to be of high quality. Two reviewers (Shi and Xie) conducted the data extraction and quality assessment independently. When necessary, the reviewers wrote to the corresponding authors for extra information or raw data. Any discrepancies between two reviewers were resolved by discussion until reaching a consensus. The final results were reviewed by a senior reviewer (Li).

### Statistical analysis

All data analyses were performed using Review Manager Version 5.3.3 (The Cochrane Collaboration, Software Update, Oxford, United Kingdom). HWE was explored with the Chi square test. ORs and 95% CIs were employed to evaluate potential associations between *IL*-*10* polymorphisms and the risk of urologic cancer. Heterogeneity between studies was assessed by using the Q test and I^2^ statistic. If probability value (*P* value) of Q test was less than 0.1 or I^2^ was greater than 50%, the random-effect model (REM) would be adopted for analyses due to the existence of significant between-study heterogeneity. Otherwise, the fixed-effect model (FEM) would be applied for analyses. Subgroup analyses were performed based on types of cancer and ethnicity of study population. Sensitivity analyses were carried out by omitting one individual study each time. Publication bias was further evaluated with funnel plots. And a *P* value of 0.05 or less was considered to be statistically significant for all analyses.

## Results

### Included studies

**Fig. 1:**
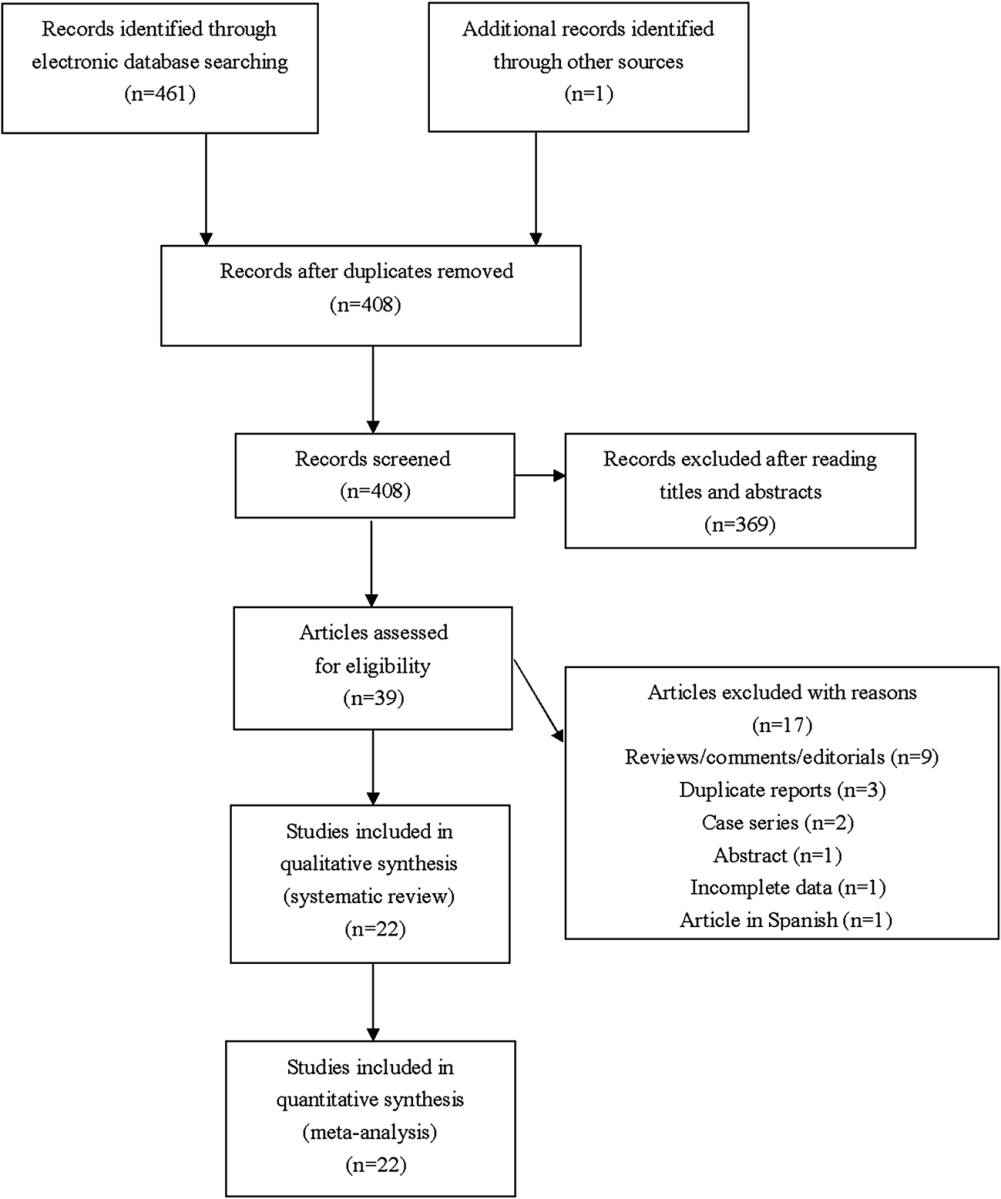
Flowchart of study selection for the present study

The literature search yielded 462 results. After exclusion of irrelevant or duplicate articles by reading titles and abstracts, 39 articles were selected for further evaluation. Among these, a total of 22 case–control studies including 8572 urologic cancer patients and 9843 control subjects met our inclusion criteria (see Fig. 1), 14/22 were about the *IL*-*10* −592C>A polymorphism, 13/22 were about the *IL*-*10* −819C>T polymorphism, and 20/22 were about the *IL*-*10* −1082A>G polymorphism. All included studies were published between 2002 and 2016. Of these, there were 16 studies of prostate cancer, 4 studies of renal cancer, and 2 studies of bladder cancer. All articles were published in English except for two in Chinese. Characteristics of studies analyzing *IL*-*10* −592C>A polymorphism were summarized in Table 1, characteristics of studies examining *IL*-*10* −819C>T polymorphism were summarized in Table 2, and characteristics of studies investigating *IL*-*10* −1082A>G polymorphism were summarized in Table 3.

**Table 1:** The characteristics of the included studies for *IL*-*10* −592 C/A polymorphism and urologic cancer risk

References	Country	Ethnicity	Case			Control			*P* value HWE	NOS score
			n	Genotypes CC/CA/AA	Alleles C/A (%)	n	Genotypes CC/CA/AA	Alleles C/A (%)		
*Prostate cancer*										
Dluzniewski et al. (2012)	USA	Mixed	442	236/171/35	72.7/27.3	442	253/168/21	76.2/23.8	0.300	7
Dwivedi et al. (2015a, b)	India	Asian	291	110/125/56	59.3/40.7	291	98/138/55	57.4/42.6	0.604	8
Eder et al. (2007)	Austria	Caucasian	547	293/219/35	73.6/26.4	545	296/216/33	74.1/25.9	0.437	8
Faupel-Badger et al. (2008)	USA	Caucasian	510	284/188/39	74.1/25.9	386	243/124/19	79.0/21.0	0.539	7
Liu et al. (2010)	China	Asian	262	34/108/120	33.6/66.4	270	28/110/132	30.7/69.3	0.477	8
VanCleave et al. (2010)	USA	African	189	72/87/30	61.1/38.9	651	251/288/112	60.7/39.3	0.063	8
Wang et al. (2009)	USA	Caucasian	255	150/95/10	77.5/22.5	255	162/84/9	80.0/20.0	0.639	7
Winchester et al. (2015)	USA	Mixed	826	495/280/51	76.9/23.1	827	511/269/47	78.1/21.9	0.146	8
Xu et al. (2005)	Sweden	Caucasian	1383	NA	74.0/26.0	780	NA	75.0/25.0	NA	8
Zabaleta et al. (2008)	USA	Mixed	546	314/191/41	75.0/25.0	529	258/228/43	70.3/29.7	0.454	8
*Renal cancer*										
Basturk et al. (2005)	Turkey	Caucasian	29	13/14/2	68.9/31.1	50	24/19/7	67.0/33.0	0.320	7
Cozar et al. (2007)	Spain	Caucasian	127	81/37/9	78.3/21.7	175	98/63/14	74.0/26.0	0.394	8
Chang et al. (2016)	Taiwan	Asian	92	4/27/61	19.0/81.0	580	24/185/371	20.1/79.9	0.877	8
*Bladder cancer*										
Chen et al. (2013)	China	Asian	400	42/140/218	28.0/72.0	400	64/168/168	27.0/73.0	*0.047*	8

*P* value of HWE test <0.05, which reached the statistically significant level are indicated in italics

*HWE* Hardy–Weinberg equilibrium, *NOS* Newcastle–Ottawa Quality Assessment Scale, *NA* not available

**Table 2:** The characteristics of the included studies for *IL*-*10* −819 C/T polymorphism and urologic cancer risk

References	Country	Ethnicity	Case			Control			*P* value HWE	NOS score
			n	Genotypes CC/CT/TT	Alleles C/T (%)	n	Genotypes CC/CT/TT	Alleles C/T (%)		
*Prostate cancer*										
Dwivedi et al. (2015a, b)	India	Asian	291	68/131/92	45.9/54.1	291	60/151/80	46.6/53.4	0.466	8
Faupel-Badger et al. (2008)	USA	Caucasian	507	283/184/40	74.0/26.0	384	244/122/18	79.4/20.6	0.585	7
Kesarwani et al. (2009)	India	Asian	159	52/68/39	54.1/45.9	259	65/125/69	49.2/50.8	0.579	8
Liu et al. (2010)	China	Asian	262	34/108/120	33.6/66.4	270	28/110/132	30.7/69.3	0.477	8
Michaud et al. (2006)	USA	Mixed	1246	716/447/83	75.4/24.6	1762	964/659/139	73.4/26.6	0.078	8
VanCleave et al. (2010)	USA	African	191	76/85/30	62.0/38.0	635	246/278/111	60.6/39.4	*0.037*	8
Winchester et al. (2015)	USA	Mixed	611	372/206/33	77.7/22.3	659	408/217/34	78.4/21.6	0.464	8
Zabaleta et al. (2008)	USA	Mixed	526	308/180/38	75.7/24.3	494	249/204/41	71.1/28.9	0.931	8
*Renal cancer*										
Basturk et al. (2005)	Turkey	Caucasian	29	13/14/2	69.0/31.0	50	24/19/7	67.0/33.0	0.320	7
Cozar et al. (2007)	Spain	Caucasian	127	81/37/9	78.3/21.7	175	98/63/14	74.0/26.0	0.394	8
Chang et al. (2016)	Taiwan	Asian	92	4/26/62	18.5/81.5	580	61/209/310	28.5/71.5	*0.005*	8
*Bladder cancer*										
Ahirwar et al. (2009)	India	Asian	214	46/103/65	45.6/54.4	385	115/165/105	51.3/48.7	*0.005*	8
Chen et al. (2013)	China	Asian	400	42/140/218	28.0/72.0	400	64/168/168	37.0/63.0	*0.047*	8

*P* value of HWE test <0.05, which reached the statistically significant level are indicated in italics

*HWE* Hardy–Weinberg equilibrium, *NOS* Newcastle–Ottawa Quality Assessment Scale, *NA* not available

**Table 3:** The characteristics of the included studies for *IL*-*10* −1082 A/G polymorphism and urologic cancer risk

References	Country	Ethnicity	Case			Control			*P* value HWE	NOS score
			n	Genotypes AA/AG/GG	Alleles A/G (%)	n	Genotypes AA/AG/GG	Alleles A/G (%)		
*Prostate cancer*										
Dluzniewski et al. (2012)	USA	Mixed	458	146/212/100	55.0/45.0	458	112/242/104	50.9/49.1	0.222	7
Faupel-Badger et al. (2008)	USA	Caucasian	509	173/251/85	58.6/41.4	382	115/194/73	55.5/44.5	0.582	7
Ianni et al. (2013)	Italy	Caucasian	171	79/74/18	45.2/54.8	96	25/43/28	48.4/51.6	0.312	7
Kesarwani et al. (2009)	India	Asian	159	69/78/12	67.9/32.1	259	111/103/45	62.7/37.3	*0.016*	8
Liu et al. (2010)	China	Asian	262	222/36/4	91.6/8.4	270	240/27/3	93.9/6.1	*0.035*	8
McCarron et al. (2002)	UK	Caucasian	247	78/113/56	54.5/45.5	223	46/120/57	47.5/52.5	0.239	7
Michaud et al. (2006)	USA	Mixed	1245	356/599/290	52.7/47.3	1763	523/857/383	54.0/46.0	0.364	8
Niu (2011)	China	Asian	98	24/56/18	53.1/46.9	88	42/44/2	72.7/27.3	*0.015*	7
Omrani et al. (2009)	Iran	Caucasian	41	5/31/5	50.0/50.0	103	16/77/10	52.9/47.1	*<0.0001*	7
VanCleave et al. (2010)	USA	African	192	22/95/75	36.3/63.7	660	92/280/288	35.2/64.8	0.074	8
Wang et al. (2009)	USA	Caucasian	255	56/130/69	47.6/52.4	257	83/117/57	55.1/44.9	0.199	7
Winchester et al. (2015)	USA	Mixed	832	206/434/192	63.0/37.0	836	204/429/203	50.1/49.9	0.447	8
Xu et al. (2005)	Sweden	Caucasian	1383	NA	53.0/47.0	780	NA	51.0/49.0	NA	8
Zabaleta et al. (2008)	USA	Mixed	541	131/277/133	49.8/50.2	523	144/280/99	54.3/45.7	0.072	8
*Renal cancer*										
Basturk et al. (2005)	Turkey	Caucasian	29	17/9/3	74.1/25.9	50	32/13/5	77.0/23.0	0.060	7
Cozar et al. (2007)	Spain	Caucasian	126	42/62/22	57.9/42.1	175	58/87/30	58.0/42.0	0.787	8
Havranek et al. (2005)	UK	Mixed	147	65/56/26	63.3/36.7	149	45/69/35	53.4/46.6	0.395	7
Chang et al. (2016)	Taiwan	Asian	92	71/16/5	85.9/14.1	580	444/107/29	85.8/14.2	*<0.0001*	8
*Bladder cancer*										
Ahirwar et al. (2009)	India	Asian	214	84/112/18	65.4/34.6	385	143/181/61	60.6/39.4	0.768	8
Chen et al. (2013)	China	Asian	400	374/25/1	96.6/3.4	400	350/48/2	93.5/6.5	0.799	8

*P* value of HWE test <0.05, which reached the statistically significant level are indicated in italics

*HWE* Hardy–Weinberg equilibrium, *NOS* Newcastle–Ottawa Quality Assessment Scale, *NA* not available

### Risk of bias in included studies

As shown in Tables 1, 2 and 3, the average NOS score of included studies was 7.59 (range from 7 to 8), suggesting that all enrolled articles were of relatively high quality. The improper selection of controls and mismatching baseline characteristics of urologic cancer cases and control subjects (age and/or ethnicity) were the major sources of biases.

### *IL*-*10* −592C>A polymorphism and urologic cancer risk

**Fig. 2:**
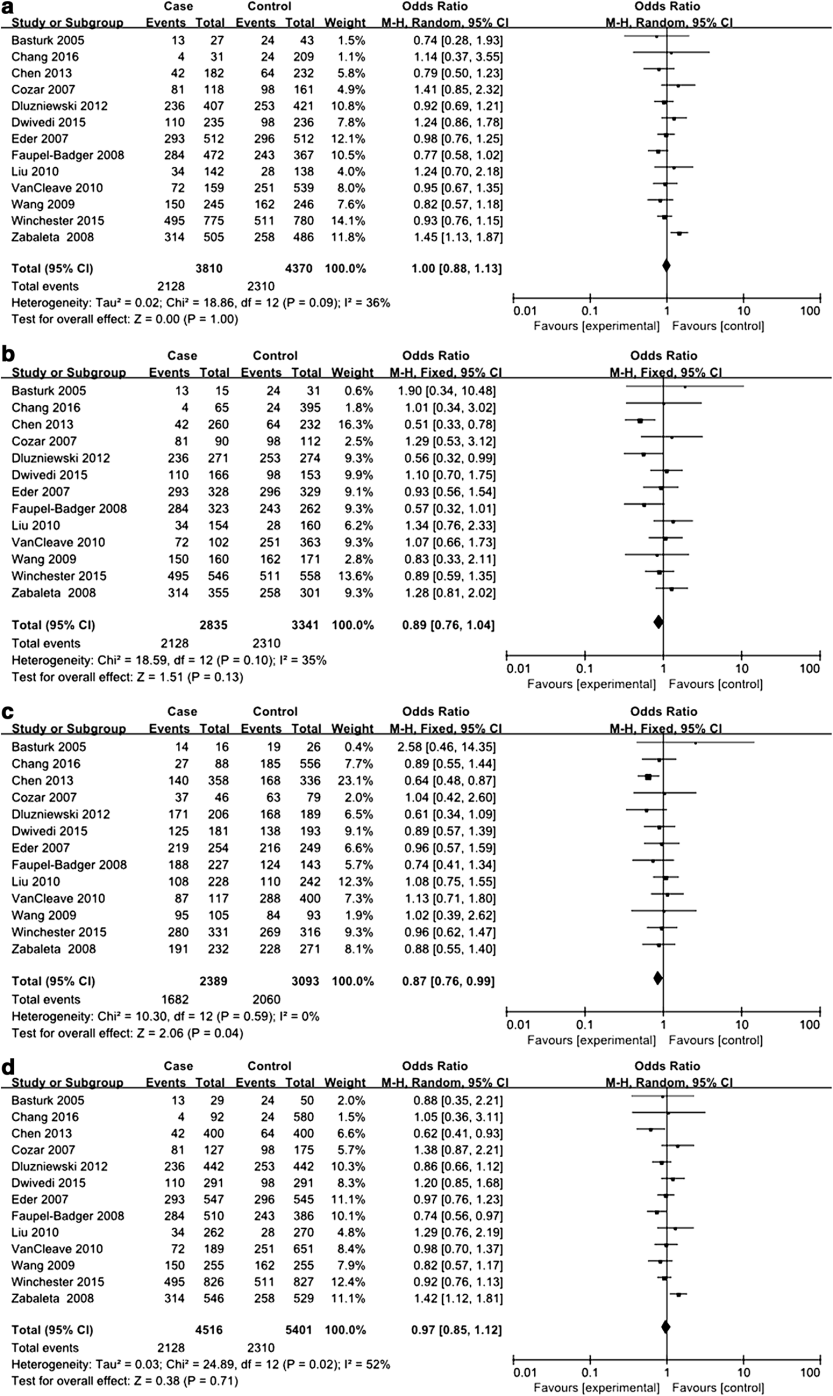
Forest plots on association between *IL*-*10* −592C>A polymorphism and urologic cancer risk. **a** Forest plot of CC versus CA for *IL*-*10* −592C>A polymorphism and urologic cancer risk is shown. **b** Forest plot of CC versus AA for *IL*-*10* −592C>A polymorphism and urologic cancer risk is shown. **c** Forest plot of CA versus AA for *IL*-*10* −592C>A polymorphism and urologic cancer risk is shown. **d** Forest plot of CC versus CA+AA for *IL*-*10* −592C>A polymorphism and urologic cancer risk is shown

**Fig. 3:**
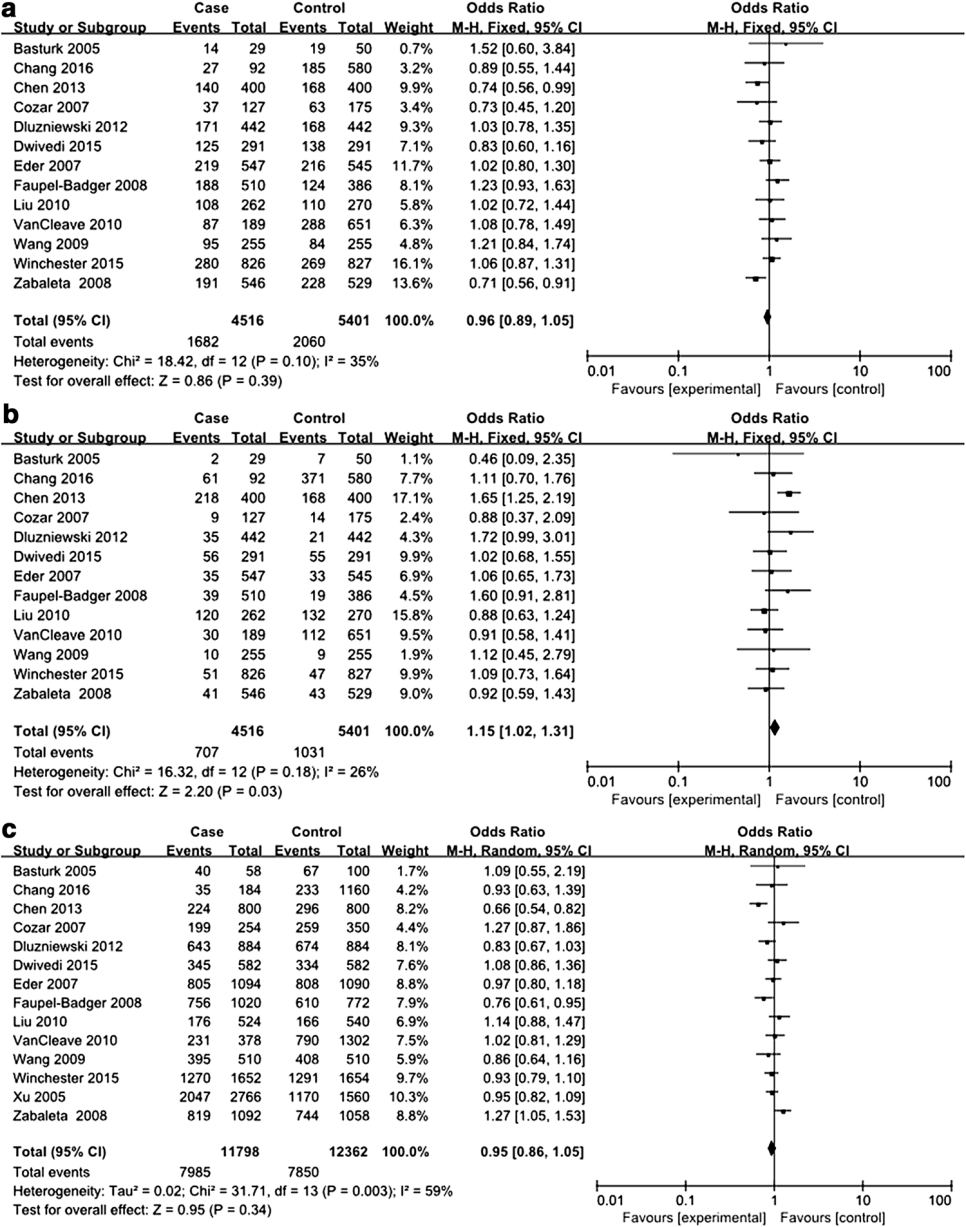
Forest plots on association between *IL*-*10* −592C>A polymorphism and urologic cancer risk. **a** Forest plot of CA versus CC+AA for *IL*-*10* −592C>A polymorphism and urologic cancer risk is shown. **b** Forest plot of AA versus CC+CA for *IL*-*10* −592C>A polymorphism and urologic cancer risk is shown. **c** Forest plot of C versus A for *IL*-*10* −592C>A polymorphism and urologic cancer risk is shown

For *IL*-*10* −592C>A polymorphism, a total of 14 studies including 5899 urologic cancer patients and 6181 control subjects were investigated (Dluzniewski et al. 2012; Dwivedi et al. 2015a, b; Eder et al. 2007; Faupel-Badger et al. 2008; Liu et al. 2010; VanCleave et al. 2010; Wang et al. 2009; Winchester et al. 2015; Xu et al. 2005; Zabaleta et al. 2008; Basturk et al. 2005; Cozar et al. 2007; Chang et al. 2016; Chen et al. 2013). HWE test for the control group of each included studies demonstrated that only 1 study deviated from HWE (see Table 1). In order to explore the association between *IL*-*10* −592C>A polymorphism and urologic cancer risk, we compared distribution of genotypes and alleles in every genetic model. For CC versus AA, CA versus AA, CA versus CC+AA, and AA versus CC+CA, between-study heterogeneity was mild, and analyses were performed with FEMs. For CC versus CA, CC versus CA+AA, and C versus A, REMs were selected due to severe between-study heterogeneity. A significant association with urologic cancer was found for *IL*-*10* −592C>A polymorphism in CA versus AA (*P* = 0.04, OR 0.87, 95% CI 0.76–0.99) and AA versus CC+CA (*P* = 0.03, OR 1.15, 95% CI 1.02–1.31) (see Figs. 2, 3).

### *IL*-*10* −819C>T polymorphism and urologic cancer risk

**Fig. 4:**
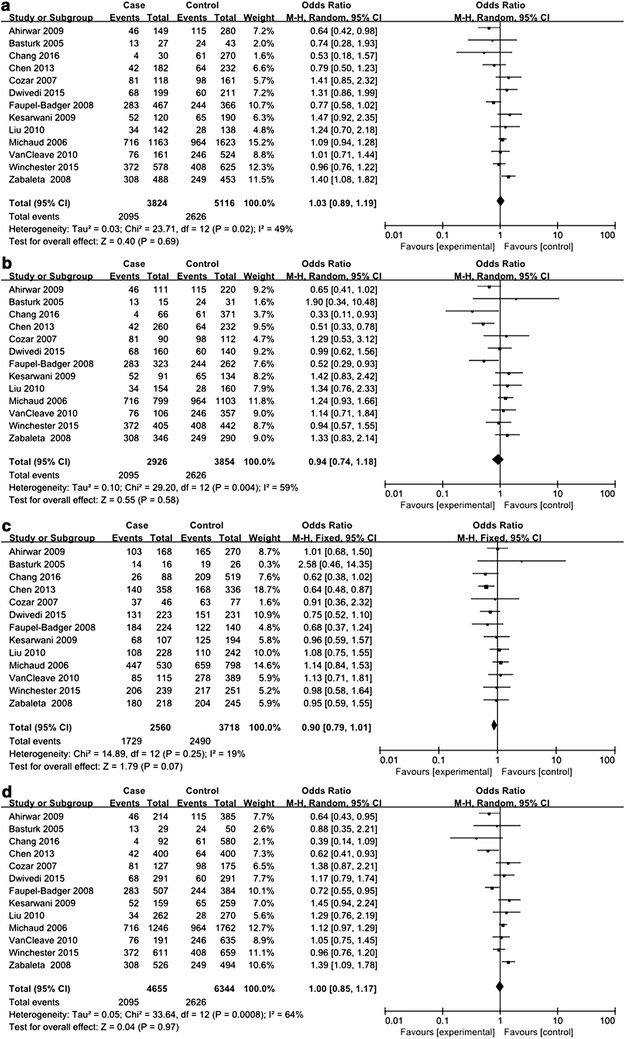
Forest plots on association between *IL*-*10* −819C>T polymorphism and urologic cancer risk. **a** Forest plot of CC versus CT for *IL*-*10* −819C>T polymorphism and urologic cancer risk is shown. **b** Forest plot of CC versus TT for *IL*-*10* −819C>T polymorphism and urologic cancer risk is shown. **c** Forest plot of CT versus TT for *IL*-*10* −819C>T polymorphism and urologic cancer risk is shown. **d** Forest plot of CC versus CT+TT for *IL*-*10* −819C>T polymorphism and urologic cancer risk is shown

**Fig. 5:**
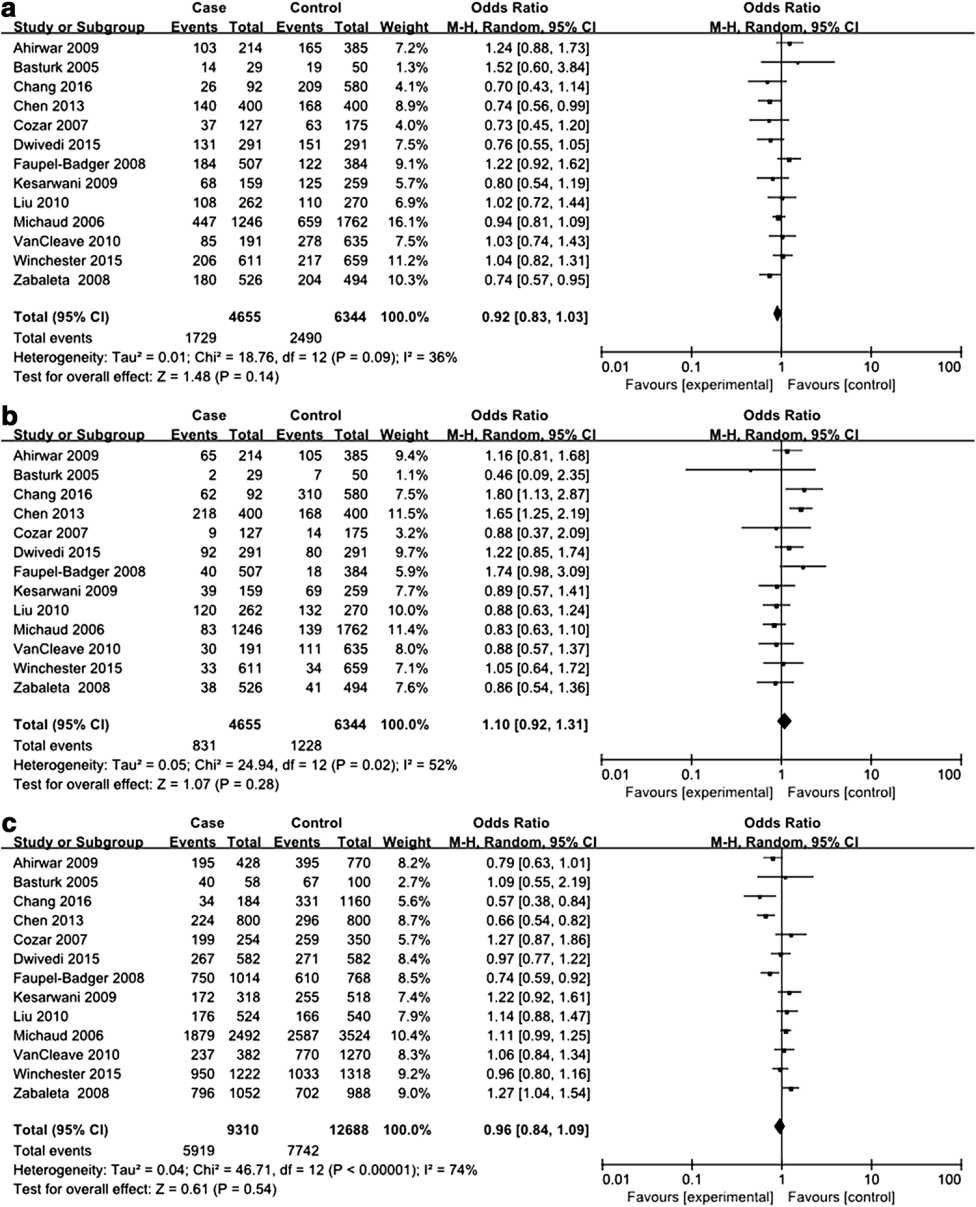
Forest plots on association between *IL*-*10* −819C>T polymorphism and urologic cancer risk. **a**. Forest plot of CT versus CC+TT for *IL*-*10* −819C>T polymorphism and urologic cancer risk is shown. **b** Forest plot of TT versus CC+CT for *IL*-*10* −819C>T polymorphism and urologic cancer risk is shown. **c** Forest plot of C versus T for *IL*-*10* −819C>T polymorphism and urologic cancer risk is shown

A total of 13 studies with 4655 cancer cases and 6344 healthy controls were enrolled to evaluate the association between *IL*-*10* −819C>T polymorphism and urologic cancer risk (Dwivedi et al. 2015a, b; Faupel-Badger et al. 2008; Liu et al. 2010; VanCleave et al. 2010; Winchester et al. 2015; Zabaleta et al. 2008; Basturk et al. 2005; Cozar et al. 2007; Chang et al. 2016; Chen et al. 2013; Kesarwani et al. 2009; Michaud et al. 2006; Ahirwar et al. 2009). HWE test for the control group of eligible studies revealed that 4 studies violated HWE (see Table 2). All genetic models were tested to detect any differences in genotypic and allelic frequencies of cases and controls. For CT versus TT, there was only trivial between-study heterogeneity, and FEM was employed for analysis. For CC versus CT, CC versus TT, CC versus CT+TT, CT versus CC+TT, TT versus CC+CT, and C versus T, between-study heterogeneity was obvious, and REMs were adopted for analyses. No significant association with urologic cancer was found for *IL*-*10* −819C>T polymorphism in any genetic models (see Figs. 4, 5).

### *IL*-*10* −1082A>G polymorphism and urologic cancer risk

**Fig. 6:**
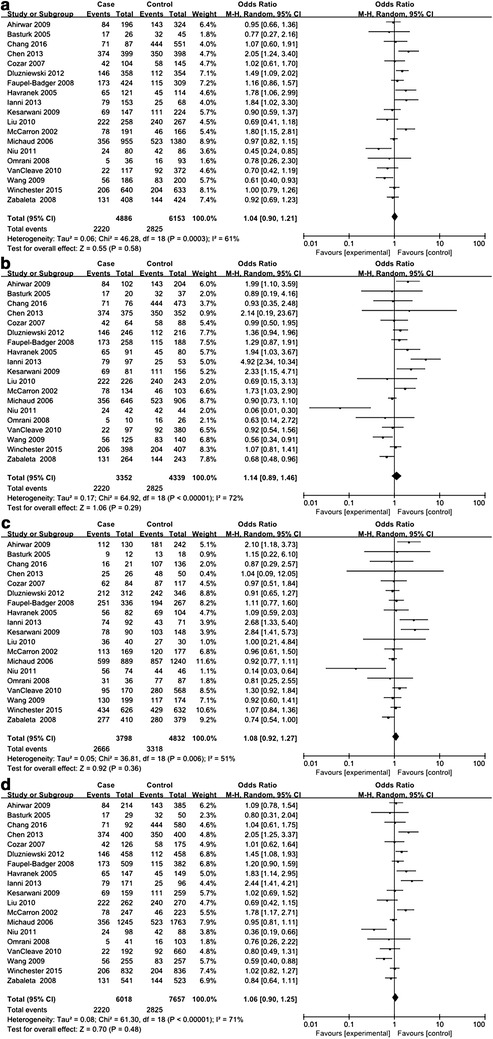
Forest plots on association between *IL*-*10* −1082A>G polymorphism and urologic cancer risk. **a** Forest plot of AA versus AG for *IL*-*10* −1082A>G polymorphism and urologic cancer risk is shown. **b** Forest plot of AA versus GG for *IL*-*10* −1082A>G polymorphism and urologic cancer risk is shown. **c** Forest plot of AG versus GG for *IL*-*10* −1082A>G polymorphism and urologic cancer risk is shown. **d** Forest plot of AA versus AG+GG for *IL*-*10* −1082A>G polymorphism and urologic cancer risk is shown

**Fig. 7:**
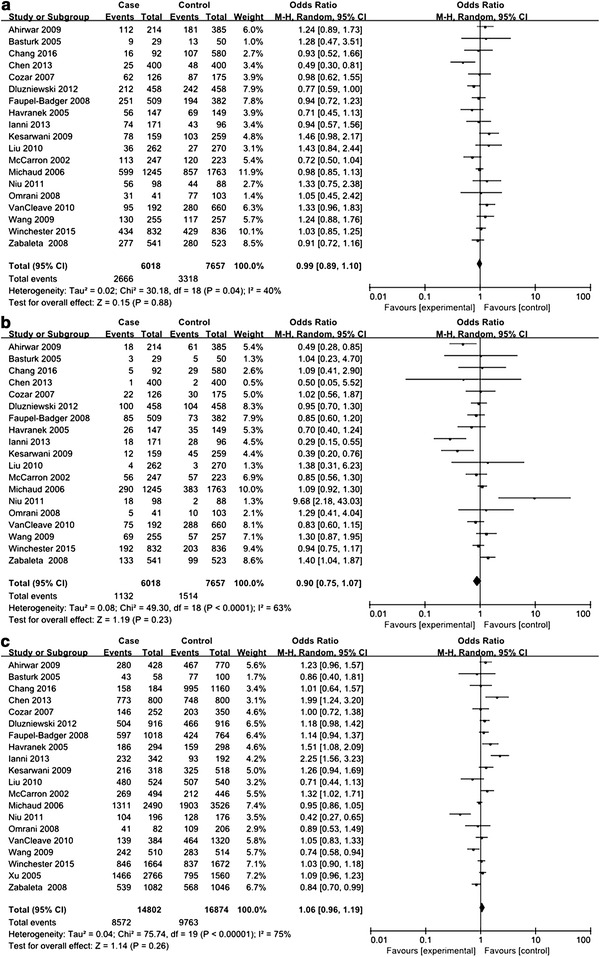
Forest plots on association between *IL*-*10* −1082A>G polymorphism and urologic cancer risk. **a** Forest plot of AG versus AA+GG for *IL*-*10* −1082A>G polymorphism and urologic cancer risk is shown. **b** Forest plot of GG versus AA+AG for *IL*-*10* −1082A>G polymorphism and urologic cancer risk is shown. **c** Forest plot of A versus G for *IL*-*10* −1082A>G polymorphism and urologic cancer risk is shown

Of the 20 included studies for *IL*-*10* −1082A>G polymorphism, there were 7401 urologic cancer patients and 8437 controls (Dluzniewski et al. 2012; Faupel-Badger et al. 2008; Liu et al. 2010; VanCleave et al. 2010; Wang et al. 2009; Winchester et al. 2015; Xu et al. 2005; Zabaleta et al. 2008; Basturk et al. 2005; Cozar et al. 2007; Chang et al. 2016; Chen et al. 2013; Kesarwani et al. 2009; Michaud et al. 2006; Ahirwar et al. 2009; Ianni et al. 2013; McCarron et al. 2002; Niu 2011; Omrani et al. 2009; Havranek et al. 2005). Deviations from HWE were found in 5 studies while the remaining 15 studies were in accordance with HWE (see Table 3). For evaluation of the association between *IL*-*10* −1082A>G polymorphism and urologic cancer risk, frequencies of genotypes and alleles in cases and control subjects were compared in every genetic model. REMs were used for all analyses on account of striking between-study heterogeneity, and no significant association was detected between *IL*-*10* −1082A>G polymorphism and urologic cancer risk (see Figs. 6, 7).

### Subgroup analysis

For *IL*-*10* polymorphisms (−592C>A, −819C>T, −1082A>G) and urologic cancer risk, subgroup analyses were performed by stratifying available data according to types of cancer and ethnicity of study population. When data were stratified by cancer types, we found that *IL*-*10* −592C>A polymorphism was significantly associated with bladder cancer risk in CC versus AA (*P* = 0.002, OR 0.51, 95% CI 0.33–0.78), CA versus AA (*P* = 0.004, OR 0.64, 95% CI 0.48–0.87), CC versus CA+AA (*P* = 0.02, OR 0.62, 95% CI 0.41–0.93), CA versus CC+AA (*P* = 0.04, OR 0.74, 95% CI 0.56–0.99), AA versus CC+CA (*P* = 0.0004, OR 1.65, 95% CI 1.25–2.19), and C versus A (*P* = 0.00001, OR 0.66, 95% CI 0.54–0.82). Besides, *IL*-*10* −819C>T polymorphism was significantly correlated with bladder cancer risk in CC versus CT (*P* = 0.03, OR 0.71, 95% CI 0.52–0.96), CC versus TT (*P* = 0.0005, OR 0.57, 95% CI 0.41–0.78), CC versus CT+TT (*P* = 0.002, OR 0.63, 95% CI 0.47–0.84), and C versus T (*P* < 0.00001, OR 0.72, 95% CI 0.61–0.84). Additionally, *IL*-*10* −1082A>G polymorphism was also significantly associated with the risk of bladder cancer in AA versus GG (*P* = 0.02, OR 2.00, 95% CI 1.13–3.55), AG versus GG (*P* = 0.01, OR 2.03, 95% CI 1.16–3.55), and GG versus AA+AG (*P* = 0.009, OR 0.49, 95% CI 0.28–0.84). When data were subsequently stratified by ethnicity, we observed a significant association with urologic cancer risk for *IL*-*10* −819C>T polymorphism in CT versus TT (*P* = 0.0009, OR 0.81, 95% CI 0.69–0.95). No any other associations were found in subgroup analyses (see Tables 4, 5, 6).

**Table 4:** Subgroup analyses for *IL*-*10* −592C>A polymorphism and urologic cancer risk

Variables	*P* value	OR (95% Cl)	*I*-square (%)	*P* for the heterogeneity
*Cancer type*				
Prostate cancer (No.^a^: 10)				
CC versus CA	0.99	1.00 (0.87–1.15)	49	0.05
CC versus AA	0.51	0.94 (0.80–1.12)	22	0.25
CA versus AA	0.37	0.93 (0.79–1.09)	0	0.85
CC versus CA+AA	0.85	0.99 (0.85–1.14)	56	0.02
CA versus CC+AA	0.97	1.00 (0.91–1.09)	36	0.13
AA versus CC+CA	0.47	1.06 (0.91–1.23)	0	0.54
C versus A	0.55	0.97 (0.89–1.07)	49	0.04
Renal cancer (No.^a^: 3)				
CC versus CA	0.35	1.21 (0.80–1.83)	0	0.50
CC versus AA	0.47	1.26 (0.67–2.36)	0	0.83
CA versus AA	0.94	0.99 (0.65–1.49)	0	0.50
CC versus CA+AA	0.30	1.23 (0.83–1.82)	0	0.66
CA versus CC+AA	0.40	0.87 (0.63–1.20)	0	0.39
AA versus CC+CA	0.98	1.00 (0.67–1.47)	0	0.56
C versus A	0.48	1.10 (0.85–1.41)	0	0.55
Bladder cancer (No.^a^: 1)				
CC versus CA	0.30	0.79 (0.50–1.23)	NA	NA
CC versus AA	*0.002*	*0.51 (0.33–0.78)*	NA	NA
CA versus AA	*0.004*	*0.64 (0.48–0.87)*	NA	NA
CC versus CA+AA	*0.02*	*0.62 (0.41–0.93)*	NA	NA
CA versus CC+AA	*0.04*	*0.74 (0.56–0.99)*	NA	NA
AA versus CC+CA	*0.0004*	*1.65 (1.25–2.19)*	NA	NA
C versus A	*0.00001*	*0.66 (0.54–0.82)*	NA	NA
*Ethnicity*				
Asian (No.^a^: 4)				
CC versus CA	0.57	1.07 (0.84–1.37)	0	0.44
CC versus AA	0.70	0.91 (0.55–1.50)	68	0.03
CA versus AA	0.05	0.82 (0.68–1.00)	40	0.17
CC versus CA+AA	0.93	0.98 (0.66–1.46)	58	0.07
CA versus CC+AA	0.06	0.85 (0.71–1.01)	0	0.58
AA versus CC+CA	0.38	1.15 (0.84–1.58)	66	0.03
C versus A	0.61	0.93 (0.70–1.23)	78	0.003
Caucasian (No.^a^: 7)				
CC versus CA	0.89	0.99 (0.86–1.13)	52	0.06
CC versus AA	0.31	0.87 (0.66–1.14)	0	0.58
CA versus AA	0.37	0.88 (0.66–1.16)	0	0.75
CC versus CA+AA	0.84	0.98 (0.80–1.20)	53	0.06
CA versus CC+AA	1.00	1.00 (0.82–1.22)	49	0.08
AA versus CC+CA	0.30	1.15 (0.88–1.51)	0	0.70
C versus A	0.31	0.96 (0.88–1.04)	35	0.16

The difference in cases and controls regarding the distributions of investigated genetic polymorphisms in certain genetic model reached the statistically significant level, which is also less than 0.05 are indicated in italics

*OR* odds ratio, *CI* confidence interval, *NA* not applicable

^a^The number of articles

**Table 5:** Subgroup analyses for *IL*-*10* −819C>T polymorphism and urologic cancer risk

Variables	*P* value	OR (95% Cl)	I-square (%)	*P* for the heterogeneity
*Cancer type*				
Prostate cancer (No.^a^: 8)				
CC versus CT	0.22	1.10 (0.95–1.27)	46	0.07
CC versus TT	0.18	1.11 (0.95–1.30)	27	0.22
CT versus TT	0.79	0.98 (0.85–1.14)	0	0.68
CC versus CT+TT	0.27	1.09 (0.94–1.27)	56	0.03
CT versus CC+TT	0.16	0.94 (0.86–1.03)	32	0.17
TT versus CC+CT	0.63	0.97 (0.84–1.11)	9	0.36
C versus T	0.44	1.05 (0.93–1.17)	58	0.02
Renal cancer (No.^a^: 3)				
CC versus CT	0.75	1.07 (0.71–1.59)	40	0.19
CC versus TT	0.77	0.85 (0.30–2.46)	60	0.08
CT versus TT	0.15	0.74 (0.49–1.11)	26	0.26
CC versus CT+TT	0.71	0.87 (0.42–1.80)	61	0.08
CT versus CC+TT	0.13	0.78 (0.57–1.08)	12	0.32
TT versus CC+CT	0.08	1.42 (0.96–2.09)	50	0.13
C versus T	0.74	0.91 (0.52–1.60)	77	0.01
Bladder cancer (No.^a^: 2)				
CC versus CT	*0.03*	*0.71 (0.52–0.96)*	0	0.51
CC versus TT	*0.0005*	*0.57 (0.41–0.78)*	0	0.45
CT versus TT	0.29	0.79 (0.51–1.23)	68	0.08
CC versus CT+TT	*0.002*	*0.63 (0.47–0.84)*	0	0.88
CT versus CC+TT	0.85	0.95 (0.58–1.57)	80	0.02
TT versus CC+CT	0.05	*1.42 (1.01–2.00)*	55	0.14
C versus T	**<0.00001**	*0.72 (0.61–0.84)*	21	0.26
*Ethnicity*				
Asian (No.^a^: 6)				
CC versus CT	0.91	0.98 (0.72–1.35)	56	0.04
CC versus TT	0.30	0.81 (0.55–1.21)	69	0.006
CT versus TT	*0.009*	*0.81 (0.69–0.95)*	34	0.18
CC versus CT+TT	0.54	0.89 (0.62–1.28)	70	0.005
CT versus CC+TT	0.05	0.87 (0.75–1.00)	36	0.17
TT versus CC+CT	0.09	1.23 (0.97–1.56)	60	0.03
C versus T	0.21	0.87 (0.70–1.08)	77	0.0005
Caucasian (No.^a^: 4)				
CC versus CT	0.81	1.04 (0.74–1.48)	64	0.04
CC versus TT	0.25	0.81 (0.57–1.16)	33	0.21
CT versus TT	0.22	0.79 (0.55–1.15)	0	0.53
CC versus CT+TT	0.89	1.03 (0.72–1.46)	68	0.02
CT versus CC+TT	0.77	0.95 (0.69–1.31)	58	0.07
TT versus CC+CT	0.33	1.22 (0.82–1.80)	13	0.33
C versus T	0.97	1.01 (0.75–1.34)	68	0.02

The difference in cases and controls regarding the distributions of investigated genetic polymorphisms in certain genetic model reached the statistically significant level, which is also less than 0.05 are indicated in italics

*OR* odds ratio, *CI* confidence interval

^a^The number of articles

**Table 6:** Subgroup analyses for *IL*-*10* −1082A>G polymorphism and urologic cancer risk

Variables	*P* value	OR (95% Cl)	*I*-square (%)	*P* for the heterogeneity
*Cancer type*				
Prostate cancer (No.^a^: 14)				
AA versus AG	0.84	0.98 (0.82–1.17)	65	0.0007
AA versus GG	0.66	1.07 (0.80–1.42)	79	<0.0001
AG versus GG	0.61	1.05 (0.87–1.27)	61	0.002
AA versus AG+GG	0.90	0.99 (0.81–1.20)	75	<0.0001
AG versus AA+GG	0.99	1.00 (0.93–1.08)	36	0.10
GG versus AA+AG	0.53	0.94 (0.76–1.15)	71	<0.0001
A versus G	0.78	1.02 (0.90–1.15)	79	<0.0001
Renal cancer (No.^a^: 4)				
AA versus AG	0.21	1.21 (0.90–1.62)	14	0.32
AA versus GG	0.23	1.28 (0.85–1.92)	0	0.41
AG versus GG	0.94	1.02 (0.68–1.51)	0	0.98
AA versus AG+GG	0.17	1.21 (0.92–1.59)	34	0.21
AG versus AA+GG	0.38	0.88 (0.67–1.16)	0	0.67
GG versus AA+AG	0.49	0.88 (0.61–1.27)	0	0.78
A versus G	0.17	1.15 (0.94–1.40)	29	0.24
Bladder cancer (No.^a^: 2)				
AA versus AG	0.42	1.37 (0.64–2.90)	83	0.01
AA versus GG	*0.02*	*2.00 (1.13–3.55)*	0	0.96
AG versus GG	*0.01*	*2.03 (1.16–3.55)*	0	0.59
AA versus AG+GG	0.23	1.46 (0.79–2.70)	76	0.04
AG versus AA+GG	0.62	0.79 (0.32–1.97)	89	0.003
GG versus AA+AG	*0.009*	*0.49 (0.28–0.84)*	0	0.99
A versus G	0.09	1.49 (0.94–2.38)	68	0.08
*Ethnicity*				
Asian (No.^a^: 6)				
AA versus AG	0.70	0.93 (0.65–1.33)	68	0.007
AA versus GG	0.90	0.95 (0.39–2.30)	76	0.0009
AG versus GG	0.74	1.14 (0.53–2.48)	67	0.01
AA versus AG+GG	0.76	0.94 (0.64–1.39)	76	0.0008
AG versus AA+GG	0.60	1.09 (0.79–1.51)	64	0.02
GG versus AA+AG	0.89	0.94 (0.42–2.12)	73	0.003
A versus G	0.99	1.00 (0.69–1.44)	83	<0.0001
Caucasian (No.^a^: 9)				
AA versus AG	0.62	1.07 (0.81–1.41)	59	0.02
AA versus GG	0.54	1.15 (0.73–1.82)	78	<0.0001
AG versus GG	0.97	1.00 (0.84–1.19)	31	0.18
AA versus AG+GG	0.59	1.09 (0.79–1.50)	73	0.0004
AG versus AA+GG	0.49	0.95 (0.83–1.09)	0	0.67
GG versus AA+AG	0.40	0.89 (0.67–1.17)	57	0.02
A versus G	0.55	1.07 (0.85–1.34)	79	<0.0001

The difference in cases and controls regarding the distributions of investigated genetic polymorphisms in certain genetic model reached the statistically significant level, which is also less than 0.05 are indicated in italics

*OR* odds ratio, *CI* confidence interval

^a^The number of articles

### Sensitivity analysis

Sensitivity analyses were carried out through removing one individual study each time. For *IL*-*10* −519C>A polymorphism, when the study performed by Dluzniewski et al. (2012), Faupel-Badger et al. (2008) or Chen et al. (2013) was excluded, the significant association with urologic cancer was no longer observed in CA versus AA, and AA versus CC+CA. For *IL*-*10* −819C>T polymorphism, when the study of Liu et al. (2010) or Michaud et al. (2006) was removed, the null association with urologic cancer in CT versus TT was altered. For *IL*-*10* −1082A>G polymorphism, however, removing any study did not impact the overall results.

### Publication bias

Potential publication bias was evaluated with funnel plots. Visual inspection of funnel plots revealed no apparent asymmetry for *IL*-*10* −592C>A, −819C>T, and −1082A>G polymorphisms. And these results indicated that significant publication bias was unlikely.

## Discussion

Urologic cancer is a major public health problem. According to a recent survey, prostate cancer, renal cancer and bladder cancer altogether accounted for 13.3% (1879,000/14090,000) new cancer cases and 7.5% (616,000/8201,000) cancer-related deaths worldwide in 2012, making the urologic cancer ranked as the second most common group of malignancies in terms of morbidity, and the third most common group of malignancies in terms of mortality (Ferlay et al. 2015).

To date, the etiologies of urologic cancer are still largely unknown in spite of extensive studies. However, it has become evident recently that multiple immunomodulatory cytokines are implicated in the process of tumor genesis (Kurzrock 2001; Smyth et al. 2004). Among these cytokines, IL-10 is a multifunctional immunological regulator mainly produced by B cells, T cells and activated monocytes/marcophages. As an important modulator of immune responses, IL-10 can be both tumor-promoting and tumor-inhibiting since it has both immunosuppressive and anti-angiogenic functions (Mocellin et al. 2005). On the one hand, the immunosuppressive property of IL-10 may suppress anti-tumor immune responses and promote tumor development. On the other hand, the anti-angiogenic property of IL-10 may inhibit microvasculature formation and tumor growth. Previous studies have found that serum level of IL-10 was significantly elevated in urologic cancer, and it was closely correlated with tumor progression and metastasis (Stearns et al. 1999; Uwatoko et al. 2002; Dwivedi et al. 2015a, b), which suggested that IL-10 may play a vital role in the development of urologic cancer.

*IL*-*10* gene is located on chromosome 1q31–32. Common promoter region polymorphisms of *IL*-*10* gene, −592C>A (rs1800872), −819C>T (rs1800871) and −1082A>G (rs1800896) were found to influence the production of IL-10 (Turner et al. 1997; Kingo et al. 2005). Consequently, it is biologically plausible that these polymorphisms may be associated with susceptibility to urologic cancer.

Recently, numerous studies have tried to explore the potential associations between *IL*-*10* polymorphisms and the risk of urologic cancer, but the results were contradicted. Thus, we conducted the present meta-analysis to solve the conflict and obtain a more conclusive result. And our overall analyses suggested that *IL*-*10* −592C>A polymorphism was significantly associated with the risk of urologic cancer in CA versus AA, and AA versus CC+CA. However, we failed to detect any significant associations with urologic cancer for *IL*-*10* −819C>T and −1082A>G polymorphisms in overall analyses. Considering the differences of carcinogenic mechanisms for each type of cancer and the importance of ethnic background in genetic investigations, stratified analyses were subsequently performed by categorizing included studies into different subgroups on the basis of types of cancer and ethnicity of study population. When data were stratified by types of cancer, we found that *IL*-*10* −592C>A, −819C>T and −1082A>G polymorphisms were all significantly associated with the risk of bladder cancer in certain genetic models. In addition, the A allele of −592C>A polymorphism and T allele of −819C>T polymorphism conferred an increased susceptibility to bladder cancer. When data were stratified by ethnicity of study population, a significant association with urologic cancer risk in Asians was detected for *IL*-*10* −819C>T polymorphism in CT versus TT. No any other significant associations between *IL*-*10* polymorphisms and urologic cancer risk were observed in subgroup analyses. For the evaluation of the heterogeneity, we found that the between-study heterogeneity remained significant in several subgroup comparisons, suggesting that differences in cancer type and ethnicity could not fully elucidate the observed inconsistent results, and other unmeasured characteristics of study participants may partially attribute to the heterogeneity between studies. Moreover, we noticed a substantial decrease of heterogeneity for *IL*-*10* −592C>A polymorphism when the study performed by Zabaleta et al. (2008) was omitted, and that for *IL*-*10* −819C>T polymorphism when the study conducted by Chen et al. (2013) was removed or that for *IL*-*10* 1082A>G polymorphism when the studies of Ianni et al. (2013) and Niu (2011) were excluded, which suggested that these studies were the major sources of the observed heterogeneity.

This study is certainly not without limitations. Firstly, the number of studies investigating the associations of certain *IL*-*10* polymorphisms with renal cancer or bladder cancer is still limited, and sample size of several included studies were obviously not sufficient, which precluded us from drawing definite conclusions. Secondly, our results were based on unadjusted estimates since the majority of included studies failed to report baseline characteristics of individuals, such as age, sex, smoking status and eating habits. And lack of analyses adjusted for these potential confounding factors may affect the reliability of our results. Thirdly, although funnel plots revealed no apparent publication bias, we still could not eliminate the possibility of publication bias since only published studies were included. Fourthly, all included studies were published in English or Chinese, therefore, maybe some qualified articles in other languages were missed. Fifthly, genetic associations of *IL*-*10* polymorphisms with urologic cancer may also be influenced by gene–gene and gene-environmental interactions. It is possible that one certain polymorphism may be associated with the risk of urologic cancer, but due to interactions with multiple genes and environmental factors, the association would no longer be observed.

## Conclusions

In conclusion, the current meta-analysis suggests that *IL*-*10* −592C>A polymorphism may implicate with urologic cancer risk. Besides, promoter region polymorphisms of *IL*-*10* may serve as potential biological markers, especially for bladder cancer. Furthermore, *IL*-*10* −819C>T polymorphism may contribute to urologic cancer susceptibility in Asians while all the three studied variants of *IL*-*10* did not relate to Caucasian urologic cancer predisposition. However, it should be pointed out that the present results concerning renal cancer and bladder cancer were based on limited number of case–control studies, and further multi-center studies with larger sample size from different populations are warranted to confirm our results. Besides, given that immunomodulating cytokines play a crucial role in regulating anti-tumor immune responses, future investigations are needed to explore the potential roles of other polymorphisms of these cytokine genes in the occurrence and development of urologic cancer.

## References

[CR1] Ahirwar D, Mandhani A, Mittal RD (2009). Interleukin-10 G-1082A and C-819T polymorphisms as possible molecular markers of urothelial bladder cancer. Arch Med Res.

[CR2] Basturk B, Yavascaoglu I, Vuruskan H (2005). Cytokine gene polymorphisms as potential risk and protective factors in renal cell carcinoma. Cytokine.

[CR3] Chang WS, Liao CH, Tsai CW (2016). The role of IL-10 promoter polymorphisms in renal cell carcinoma. Anticancer Res.

[CR4] Chen ZG, Zhou W, Dai MJ, Wu ZG, Jin R (2013). Association between the interaction polymorphisms of interleukin-10 and smoking on patients with bladder cancer risk from a case–control study. Zhonghua Liu Xing Bing Xue Za Zhi.

[CR5] Cozar JM, Romero JM, Aptsiauri N (2007). High incidence of CTLA-4 AA (CT60) polymorphism in renal cell cancer. Hum Immunol.

[CR6] Dluzniewski PJ, Wang MH, Zheng SL (2012). Variation in IL10 and other genes involved in the immune response and in oxidation and prostate cancer recurrence. Cancer Epidemiol Biomark Prev.

[CR7] Dwivedi S, Goel A, Khattri S (2015). Genetic variability at promoters of IL-18 (pro-) and IL-10 (anti-) inflammatory gene affects susceptibility and their circulating serum levels: an explorative study of prostate cancer patients in North Indian populations. Cytokine.

[CR8] Dwivedi S, Goel A, Mandhani A (2015). Functional genetic variability at promoters of pro-(IL-18) and anti-(IL-10) inflammatory affects their mRNA expression and survival in prostate carcinoma patients: five year follow-up study. Prostate.

[CR9] Eder T, Mayer R, Langsenlehner U (2007). Interleukin-10 [ATA] promoter haplotype and prostate cancer risk: a population-based study. Eur J Cancer.

[CR10] Eskdale J, Kube D, Tesch H, Gallagher G (1997). Mapping of the human IL10 gene and further characterization of the 5′ flanking sequence. Immunogenetics.

[CR11] Faupel-Badger JM, Kidd LC, Albanes D, Virtamo J, Woodson K, Tangrea JA (2008). Association of IL-10 polymorphisms with prostate cancer risk and grade of disease. World J Urol.

[CR12] Ferlay J, Soerjomataram I, Dikshit R (2015). Cancer incidence and mortality worldwide: sources, methods and major patterns in GLOBOCAN 2012. Int J Cancer.

[CR13] Havranek E, Howell WM, Fussell HM, Whelan JA, Whelan MA, Pandha HS (2005). An interleukin-10 promoter polymorphism may influence tumor development in renal cell carcinoma. J Urol.

[CR14] Ianni M, Porcellini E, Carbone I (2013). Genetic factors regulating inflammation and DNA methylation associated with prostate cancer. Prostate Cancer Prostatic Dis.

[CR15] Jiang W, Sun G, Xiong J, Xi X, Shi Z (2014). Association of CYP1B1 L432V polymorphism with urinary cancer susceptibility: a meta-analysis. Diagn Pathol.

[CR16] Kesarwani P, Ahirwar DK, Mandhani A (2009). IL-10 1082 G>A: a risk for prostate cancer but may be protective against progression of prostate cancer in North Indian cohort. World J Urol.

[CR17] Kingo K, Ratsep R, Koks S, Karelson M, Silm H, Vasar E (2005). Influence of genetic polymorphisms on interleukin-10 mRNA expression and psoriasis susceptibility. J Dermatol Sci.

[CR18] Kurzrock R (2001). Cytokine deregulation in cancer. Biomed Pharmacother.

[CR19] Liu J, Song B, Bai X (2010). Association of genetic polymorphisms in the interleukin-10 promoter with risk of prostate cancer in Chinese. BMC Cancer.

[CR20] McCarron SL, Edwards S, Evans PR (2002). Influence of cytokine gene polymorphisms on the development of prostate cancer. Cancer Res.

[CR21] Michaud DS, Daugherty SE, Berndt SI (2006). Genetic polymorphisms of interleukin-1B (IL-1B), IL-6, IL-8, and IL-10 and risk of prostate cancer. Cancer Res.

[CR22] Mocellin S, Marincola FM, Young HA (2005). Interleukin-10 and the immune response against cancer: a counterpoint. J Leukoc Biol.

[CR23] Niu WQ (2011). The study on the association between the IL-10 promoter genetic polymorphisms and prostate cancer in Hubei Hans population. Int J Lab Med.

[CR24] Omrani MD, Bazargani S, Bageri M (2009). Interlukin-10, interferon-g and tumor necrosis factor—a genes variation in prostate cancer and benign prostatic hyperplasia. Curr Urol.

[CR25] Siegel R, Ma J, Zou Z, Jemal A (2014). Cancer statistics, 2014. CA Cancer J Clin.

[CR26] Smyth MJ, Cretney E, Kershaw MH, Hayakawa Y (2004). Cytokines in cancer immunity and immunotherapy. Immunol Rev.

[CR27] Stearns ME, Rhim J, Wang M (1999). Interleukin 10 (IL-10) inhibition of primary human prostate cell-induced angiogenesis: IL-10 stimulation of tissue inhibitor of metalloproteinase-1 and inhibition of matrix metalloproteinase (MMP)-2/MMP-9 secretion. Clin Cancer Res.

[CR28] Turner DM, Williams DM, Sankaran D, Lazarus M, Sinnott PJ, Hutchinson IV (1997). An investigation of polymorphism in the interleukin-10 gene promoter. Eur J Immunogenet.

[CR29] Uwatoko N, Tokunaga T, Hatanaka H (2002). Expression of interleukin-10 is inversely correlated with distant metastasis of renal cell carcinoma. Int J Oncol.

[CR30] VanCleave TT, Moore JH, Benford ML (2010). Interaction among variant vascular endothelial growth factor (VEGF) and its receptor in relation to prostate cancer risk. Prostate.

[CR31] Wang MH, Helzlsouer KJ, Smith MW (2009). Association of IL10 and other immune response-and obesity-related genes with prostate cancer in CLUE II. Prostate.

[CR32] Winchester DA, Till C, Goodman PJ (2015). Variation in genes involved in the immune response and prostate cancer risk in the placebo arm of the prostate cancer prevention trial. Prostate.

[CR33] Xu J, Lowey J, Wiklund F (2005). The interaction of four genes in the inflammation pathway significantly predicts prostate cancer risk. Cancer Epidemiol Biomark Prev.

[CR34] Zabaleta J, Lin HY, Sierra RA (2008). Interactions of cytokine gene polymorphisms in prostate cancer risk. Carcinogenesis.

[CR35] Zhang R, Wang J, Yang R (2014). Effects of Pro12Ala polymorphism in peroxisome proliferator-activated receptor-γ2 gene on metabolic syndrome risk: a meta-analysis. Gene.

